# Upregulation of transmitter release probability improves a conversion of synaptic analogue signals into neuronal digital spikes

**DOI:** 10.1186/1756-6606-5-26

**Published:** 2012-08-01

**Authors:** Jiandong Yu, Hao Qian, Jin-Hui Wang

**Affiliations:** 1 State Key Lab for Brain and Cognitive Sciences, Institute of Biophysics, Chinese Academy of Sciences, Beijing, China 100101; 2 Graduate School of Chinese Academy of Sciences, Beijing, China 100049

**Keywords:** Synapse, Neuron, Release probability, Action potential and Neuronal encoding

## Abstract

Action potentials at the neurons and graded signals at the synapses are primary codes in the brain. In terms of their functional interaction, the studies were focused on the influence of presynaptic spike patterns on synaptic activities. How the synapse dynamics quantitatively regulates the encoding of postsynaptic digital spikes remains unclear. We investigated this question at unitary glutamatergic synapses on cortical GABAergic neurons, especially the quantitative influences of release probability on synapse dynamics and neuronal encoding. Glutamate release probability and synaptic strength are proportionally upregulated by presynaptic sequential spikes. The upregulation of release probability and the efficiency of probability-driven synaptic facilitation are strengthened by elevating presynaptic spike frequency and Ca^2+^. The upregulation of release probability improves spike capacity and timing precision at postsynaptic neuron. These results suggest that the upregulation of presynaptic glutamate release facilitates a conversion of synaptic analogue signals into digital spikes in postsynaptic neurons, i.e., a functional compatibility between presynaptic and postsynaptic partners.

## Introduction

In the neuronal networks, the information flows in a sequence of action potentials, synaptic transmission and action potentials [1,2]. The digital spikes and analogue synaptic responses constitute the brain codes for well-organized behaviors and cognition. The conversions of digital-to-analogue and analogue-to-digital signals are fulfilled by the interactions of the synapses and neurons [3-13]. In the processing of signal conversion, how presynaptic sequential spikes influence synapse dynamics and in turn regulate postsynaptic spike encoding remains to be addressed in a quantitative manner. The revelation of these regulations helps understanding how numerous synapses are convergent onto each neuron and drive it to encode digital spikes precisely.

The quantitative values of synapse dynamics are influenced by the probability of transmitter release, the number of release sites and the content of released transmitters from presynaptic terminals [14-24], as well as by the number and responsiveness of postsynaptic receptors [25]. A study revealed an essential role of synaptic patterns affected by postsynaptic receptors in spike encodings [12]. It remains unclear how these presynaptic factors regulate the dynamics of individual synapses, the signal integration from numerous synapses and the encoding of digital spikes at postsynaptic neurons. Here, we present our study how glutamate release probability regulates synapse dynamics and neuron encoding in a preparation from cortical pyramidal neurons to GABAergic neurons.

## Results

Information flow in neural network is a sequence of action potentials at presynaptic neurons, signal transmission at synapses and action potentials at postsynaptic cells, i.e., sequential conversions of digital-to-analogue and analogue-to-digital signals. In the analyses of their quantitative correlation, we focused on investigating how presynaptic sequential spikes influenced synapse dynamics, such as glutamate release probability and synaptic facilitation, as well as how the release probability regulated postsynaptic spike encodings.

In terms of the strategies to address these questions, we first measured the activities of unitary synapses by recording the pairs of pyramidal-to-GABAergic neurons in cortical slices. To manipulate glutamate release probability, we changed the levels of presynaptic Ca^2+^ by infusing adenophostin-A (100 nM) or BAPTA (1 mM) into the recorded pyramidal neurons (Methods). Adenophostin-A was a specific and potent agonist of IP3Rs that induced Ca^2+^ releases from intracellular stores [26] to elevate cytoplasm Ca^2+^, and BAPTA was a chelater of Ca^2+ ^[27]. After collected the data about the influences of presynaptic Ca^2+^ on release probability and synaptic patterns, we applied a computational simulation to integrate the waveforms from numerous synapses under the conditions of different release probabilities. Finally, we injected these integrated current waveforms into postsynaptic neurons and analyzed how the glutamate release probability influenced postsynaptic spike encoding.

### Presynaptic sequential spikes upregulate transmitter release probability

**Figure 1:**
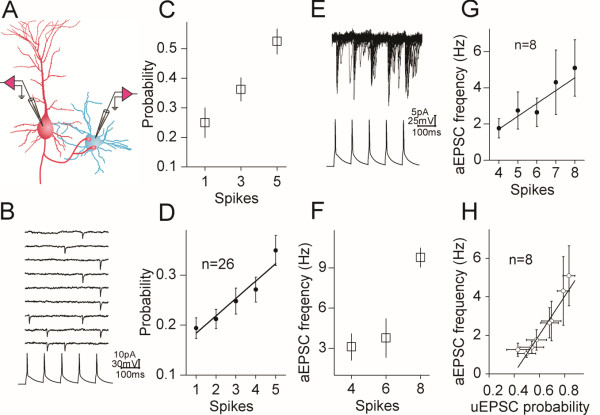
**The probability of releasing glutamates increases during sequential presynaptic spikes in a linearly correlated manner. A) **shows a diagram for the pair-recording of uEPSCs at unitary synapses from a pyramidal neuron to a GABAergic cell. **B) **shows synaptic responses evoked by five sequential spikes in a low glutamate release probability. Top traces show the superimposed traces of uEPSCs at a synapse, and a trace in bottom is sequential spikes evoked in presynaptic neuron. **C) **illustrates the averaged data of spikes vs. their corresponding release probability at this synapse. **D) **illustrates the averaged data of spikes vs. their corresponding release probability at other synapses (n = 26). **E) **shows the superimposed traces of uEPSCs and subsequent aEPSCs induced by sequential spikes 4 ~ 8 at a synapse. **F) **illustrates the averaged data of spikes vs. their corresponding aEPSCs frequency at this synapse. **G) **shows the averaged data of spikes versus their corresponding aEPSCs frequency at other synapses (n = 8). **H) **shows a plot of linear correlation between uEPSC1 ~ 8 amplitudes and their corresponding aEPSCs frequency (n = 8). Lines in E~F illustrate linear dynamical fitting.

The relationships between sequential spikes and transmitter release probability were studied at glutamatergic synapses by pair-recordings. Spikes were induced at presynaptic pyramidal neurons, and unitary excitatory postsynaptic currents (uEPSC) were recorded at GABAergic neurons (Figure 1A). In order to quantify glutamate release probability based on uEPSCs, we should rule out the saturation of vesicle release probability during sequential spikes and the effect of releasing multiple vesicles on uEPSCs. Our strategy is to analyze the synapses in low release probability (*p* < 0.25), a physiological state *in vivo *[16], evoked by the first one of sequential spikes. Under this condition, glutamate release probabilities by subsequent spikes are likely read out in a range of 0 ~ 1. Moreover, as the synchronous incidence of independent events is equal to the multiplication of their probabilities, a low probability reduces the chance of synchronously releasing two vesicles, and its values can be defined as the probability of releasing individual vesicles.

The influence of presynaptic sequential spikes on glutamate release probability is showed in Figure 1. uEPSCs (top traces in 1B) were induced by five spikes (a bottom trace). The probability of releasing glutamates at this unitary synapse increases during sequential spikes (Figure 1C). The averaged values of probabilities for evoking uEPSC1 ~ 5 are 0.19 ± 0.02, 0.21 ± 0.019, 0.25 ± 0.026, 0.27 ± 0.025 and 0.35 ± 0.03 (Figure 1D, n = 26). The increments of release probability by sequential spikes were also seen at the synapses with medium probability. Therefore, glutamate release probability is elevated by presynaptic sequential spikes. This conclusion is consistent with the views from studying other synapses [24].

We also analyzed asynchronous (a)EPSCs that were associated with uEPSCs after spikes 4 at the synapses in high probability (Figure 1E ~ H). aEPSCs were presumably evoked by glutamates released from single vesicles [28-30]. The superimposed traces in Figure 1E show aEPSC4 ~ 8 after their correspondent uEPSCs. aEPSCs frequency increases at this synapse during the sequential spikes (Figure 1F). The values for aEPSC4 ~ 8 frequency are 1.76 ± 0.53, 2.75 ± 1.0, 2.65 ± 0.8, 4.3 ± 1.78 and 5.1 ± 1.6 Hz at these synapses (Figure 1G, n = 8). It is noteworthy that the probabilities of evoked glutamate release and the frequencies of their corresponding asynchronous release are linearly correlated (Figure 1H, r^2^ = 0.92). This finding indicates that two types of glutamate release share similar mechanisms, as well as grants that sequential spikes upregulate glutamate release probability.

**Figure 2:**
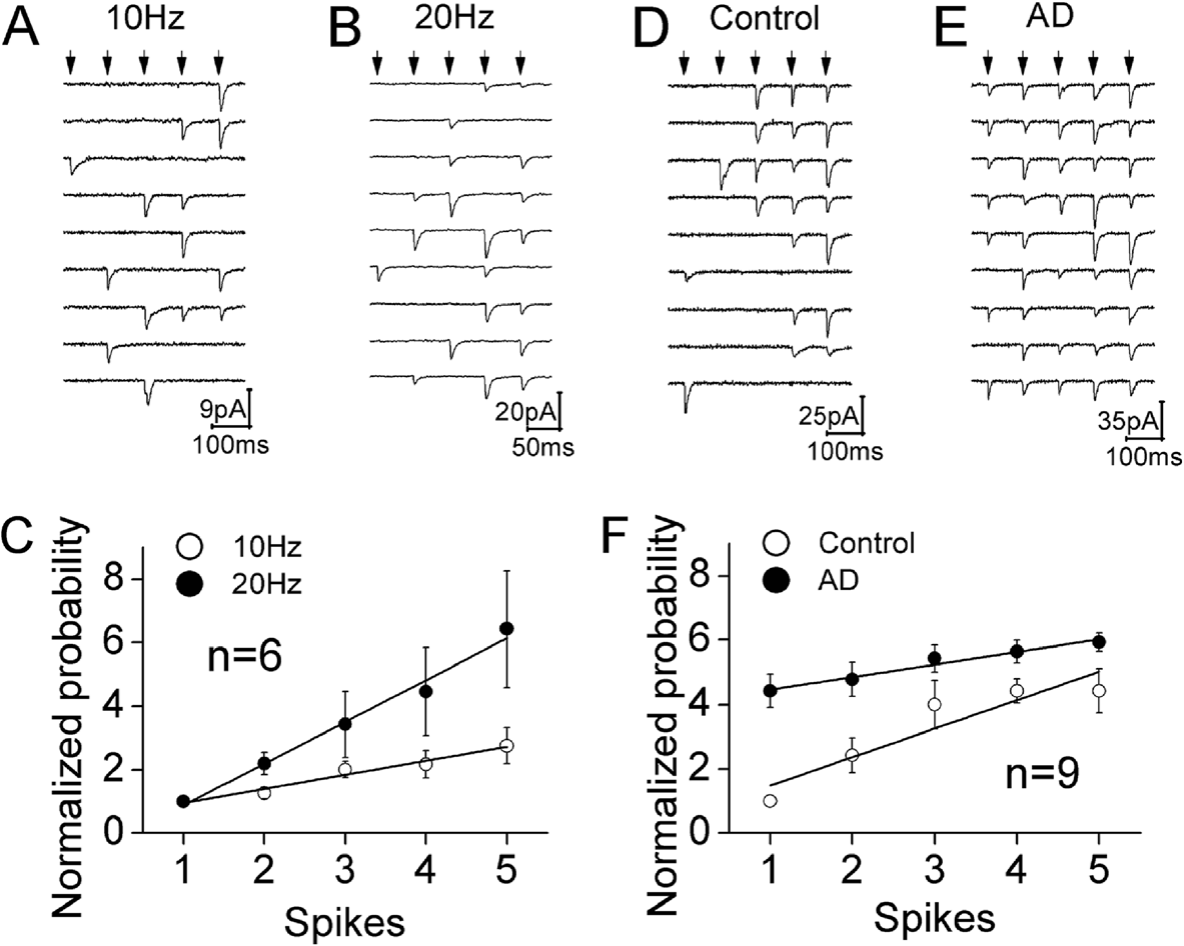
**The probability of releasing glutamates is up-regulated by spike frequency and presynaptic Ca**^**2+**^**. A) **shows the waveforms of uEPSC1 ~ 5 induced by five sequential spikes (arrows) with 10 Hz in frequency at a synapse. Calibration bar, 9 pA/100 ms. **B) **shows the waveforms of uEPSC1 ~ 5 induced by five sequential spikes (arrows) with 20 Hz in frequency at this synapse. Calibration bar, 20 pA/50 ms. **C) **shows the comparisons of the normalized probabilities of releasing glutamates by five spikes in frequencies at 10 Hz (opened symbols) vs. 20 Hz (filled symbols; n = 6). **D) **shows the waveforms of uEPSC1 ~ 5 induced by five sequential spikes (arrows) under the control at a synapse. Calibration bar, 25 pA/100 ms. **E) **shows the waveforms of uEPSC1 ~ 5 induced by five sequential spikes (arrows) under adenophostin-A (AD) infusion at a synapse. Calibration bar, 35 pA/100 ms. **F) **shows the comparisons of the normalized probabilities of releasing glutamates by five spikes under control (opened symbols) vs. AD infusion (filled symbols; n = 9). Lines in **C **and **F **illustrate linear dynamical fitting.

The upregulation of glutamate release probability was subsequently studied by presynaptic manipulations (Figure 2) to make sure this phenomenon and its underlying mechanisms. As transmitter release probability was controlled by presynaptic Ca^2+ ^[24], we changed the levels of presynaptic Ca^2+^. Presynaptic Ca^2+^ level was lowered by presynaptic infusions of BAPTA, a Ca^2+^ chelator, and elevated by raising spike frequency or infusing 100 nM adenophostin-A. It is noteworthy that these two approaches elevate presynaptic Ca^2+^ through different ways. Spike frequency changed the levels of residue Ca^2+ ^[24], whereas adenophostin-A elevated the basal level of presynaptic Ca^2+ ^[26]. It is noteworthy that release probability changes are analyzed and presented as normalized probability, in which the probability in initial response (P1) are defined as one, and the probabilities under other conditions are calculated as probability ratio (Pn/P1; also see Methods).

Figure 2A ~ B illustrates the change of uEPSC probability by raising presynaptic spike frequency from 10 Hz (2A) to 20 Hz (2B) at a unitary synapse. Their quantitative data in Figure 2C (n = 6) show that the values of normalized probabilities (Pn/P1) are 1 ± 0, 1.27 ± 0.18, 2.0 ± 0.25, 2.2 ± 0.44 and 2.76 ± 0.56 for spike frequency at 10 Hz (open symbols), and those values are 1 ± 0, 2.2 ± 0.35, 3.43 ± 1, 4.46 ± 1.38 and 6.43 ± 1.84 for spike frequency at 20 Hz (filled ones; p < 0.05 for P2 ~ P5). The release probabilities versus sequential spikes under these two conditions are linearly correlated (r^2^ = 0.95 and slope = 0.445 for 10 Hz spikes; r^2^ = 0.98 and slope = 1.3 for 20 Hz ones). The slope of release probability increment is increased by elevating spike frequency. This result supports the indication that presynaptic sequential spikes upregulate transmitter release probability (Figure 1) via residue Ca^2+ ^[24].

Figure 2E ~ F shows the influence of elevating presynaptic Ca^2+^ on release probability. A rise of presynaptic Ca^2+^ by adenophostin-A appears to increase release probability (2D ~ E). The quantitative data in Figure 2F (n = 9) show that the normalized probabilities are 1 ± 0, 2.43 ± 0.54, 4.0 ± 0.76, 4.43 ± 0.4 and 4.34 ± 0.7 under the control, and those values are 4.43 ± 0.52, 4.79 ± 0.53, 5.43 ± 0.42, 5.63 ± 0.35 and 5.93 ± 0.28 under adenophostin-induced presynaptic Ca^2+^ elevation (p < 0.01 for P1 ~ P5). Therefore, the elevation of presynaptic Ca^2+^ enhances transmitter release probability. Release probabilities vs. spikes are linearly correlated (r^2^ = 0.9 for control; r^2^ = 0.92 for Ca^2+^ elevation). The data by raising presynaptic Ca^2+^ provide a direct evidence for a theory that the presynaptic Ca^2+^ upregulates glutamate release probability.

**Figure 3:**
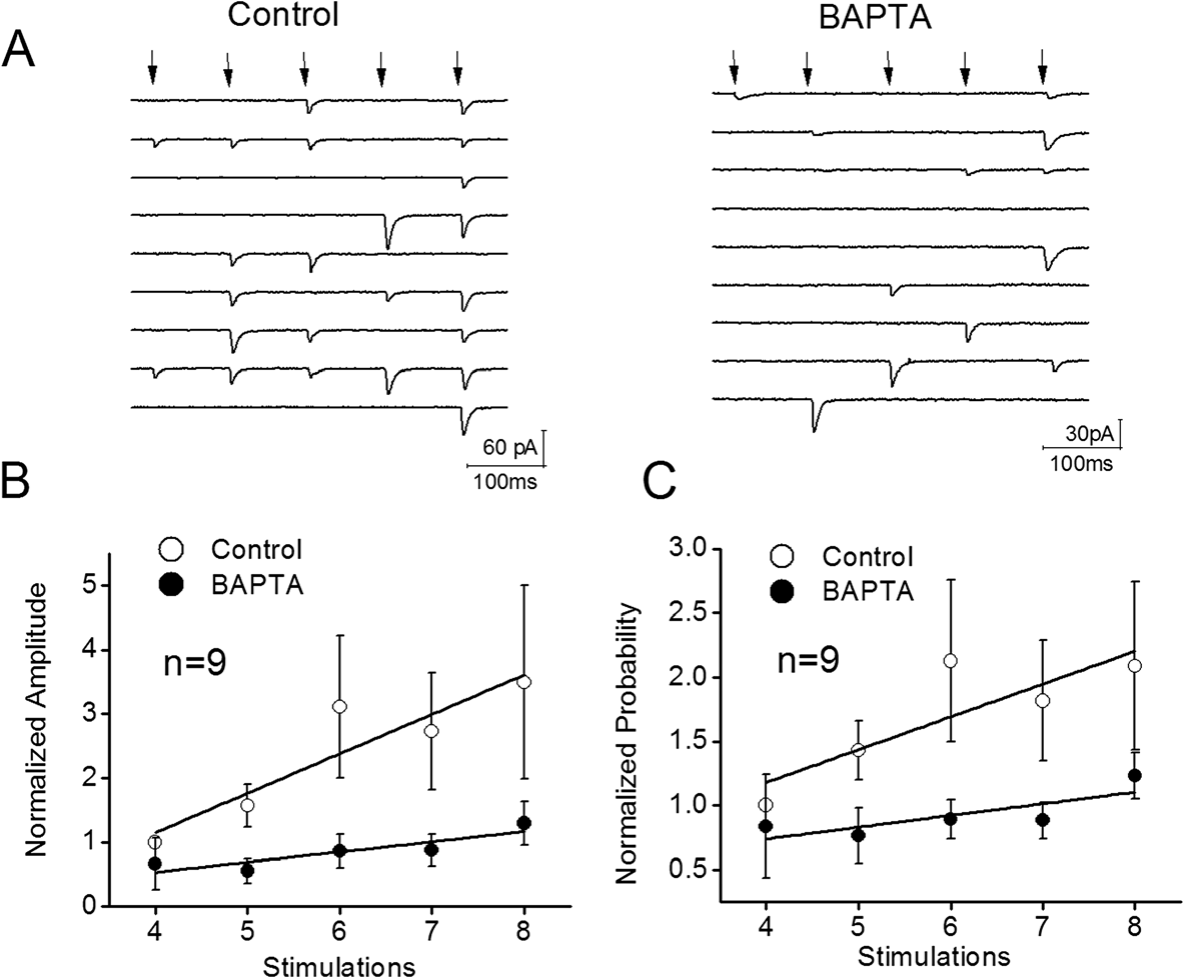
**The reduction of presynaptic Ca**^**2+ **^**by infusing BAPTA attenuates the increment of release probability of glutamate release and the facilitation of synaptic transmission. A) **Left panel shows the waveforms of uEPSC4 ~ 8 induced by sequential spikes (pointed by arrows) at a synapse under control. Calibration bar, 60 pA/100 ms. Right panel shows the waveforms of uEPSC4 ~ 8 induced by these sequential spikes (arrows) at this synapse under BAPTA infusion. Calibration bar, 30 pA/100 ms. **B) **shows the normalized uEPSC amplitudes under the conditions of control (opened symbols) versus BAPTA infusion (filled symbols; n = 10). **C) **shows the normalized probabilities of releasing glutamate under the conditions of control (opened symbols) versus BAPTA infusion (filled symbols; n = 10). It is noteworthy that the experiments were done at the synapses with low probability, such that uEPSC4 ~ 8 were analyzed. Lines in **B **and **C **illustrate linear dynamical fitting.

On the other hand, a reduction of presynaptic Ca^2+^ by presynaptically infusing 1 mM BAPTA attenuates the increment of release probability of glutamate release and the facilitation of synaptic transmission (Figure 3).

In terms of physiological significance for the increment of glutamate release probability, we further investigated the relationships between presynaptic release probability and synaptic facilitation as well as between release probability and spike encoding at postsynaptic neurons. Although everyone can predict that the increment of transmitter release probability facilitates synaptic transmission and subsequent spike encoding, the evidence based on quantitative analyses remains to be lack.

### The quantitative influences of glutamate release probability on synapse dynamics

It was well known that the increases of transmitter release probability at the CNS synapses facilitated synaptic transmission [19,21,22,24,31-33]. We focused on studying quantitative correlation between release probability and synaptic facilitation. If an increment of release probability leads to synaptic facilitation, they should be correlated proportionally under the different conditions, such as the changes in presynaptic spike frequency and Ca^2+^ level. We analyzed their correlations at the synapses with low, medium and high release probabilities. It is noteworthy that uEPSC amplitudes are averaged from synaptic responses and failures, which is better to show a role of release probability in synaptic facilitation.

**Figure 4:**
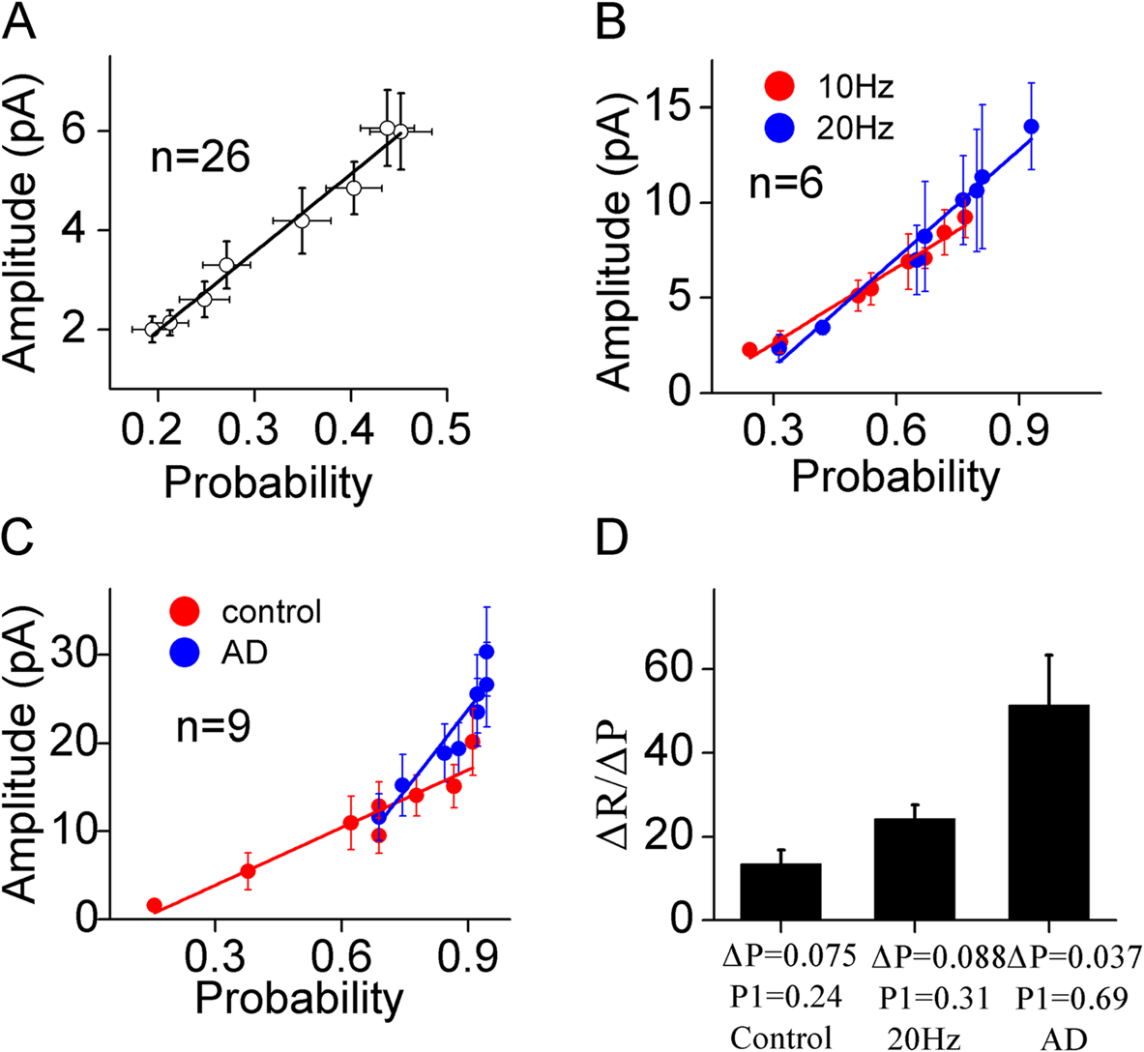
**Presynaptic Ca**^**2+ **^**enhances an efficiency of probability-driven facilitation. A) **shows a linear correlation between the probability of releasing glutamate and the amplitude of unitary synaptic responses (uEPSCs). **B) **shows the comparisons of linear correlations between release probability and uEPSCs induced by eight spikes at 10 Hz (red symbols) and 20 Hz (blues, n = 6). The efficiency of probability-driven facilitation is high when presynaptic spikes are 20 Hz. **C) **shows the comparisons of linear correlations between release probability and uEPSCs induced by eight spikes under control (red-filled symbols) and adenophostin-A infusion (blues, n = 9). The efficiency of probability-driven facilitation is high when presynaptic Ca^2+ ^is elevated by AD infusion. **D) **A plot shows the ratios of changes in uEPSCs to release probability vs. the levels of release probability, i.e., the higher release probability is, the higher efficiency of probability-driven facilitation. Lines in **A ~ C **illustrate linear dynamical fitting.

Relationships between glutamate release probabilities and uEPSC amplitudes under different conditions are illustrated in Figure 4. The release probabilities and synaptic strengths are proportionally and linearly correlated at the synapses with a low probability (r^2^ = 0.98, slope = 15.77 in 4A). Figure 4B shows the linearly correlated increases in release probability and synaptic strength at the synapses in medium probability under the conditions of 10 Hz presynaptic spikes (red symbols and line; r^2^ = 0.97, slopes = 13.2) and 20 Hz spikes (blues; r^2^ = 0.97, slope = 19). Moreover, the release probabilities and uEPSCs are proportionally linearly correlated under the conditions of control (red symbols in Figure 4C; r^2^ = 0.91, slopes = 21.8) and adenophostin-induced presynaptic Ca^2+^ elevation (blues; r^2^ = 0.87, slope = 61). Thus, the upregulation of transmitter release probability and the facilitation of synaptic transmission are naturally associated.

As the elevations of presynaptic Ca^2+^ raises the slopes of linear correlations between release probability and synaptic facilitation (Figure 4B ~ C), high release probability may more efficiently induce synaptic facilitation. To strengthen this indication, we analyzed the ratios of net change in facilitation (ΔR = Rn-(Rn-1)) to net change in probability (ΔP = Pn-(Pn-1)), which denotes the efficiency of release probability to drive synaptic facilitation. Figure 4D illustrates that the ratios of net synaptic facilitation to net release probability increase (ΔR/ΔP) are 13.5 under control (P1 = 0.24), 26 under an increased spike frequency (P1 = 0.31) and 51.3 under the elevated presynaptic Ca^2+^ level (P1 = 0.69). Thus, high release probability more efficiently boosts synaptic facilitation through recruiting more release sites.

Based on the indications above, i.e., the increase of presynaptic transmitter release probability facilitates synaptic transmission, we subsequently examined how the facilitation of release probability and synaptic transmission regulated spike encodings in postsynaptic neurons. This subject was studied by the computational simulation, as it is very challenge to study how numerous synapses convergent on a neuron drive its spike encoding by multiple whole-cell recordings on many presynaptic neurons and a postsynaptic neuron that are synaptically connected ([12,30]; Methods).

### The influences of glutamate release probability on spike encoding at postsynaptic neurons

Many synapses are convergent onto a neuron, and the analogue signals integrated from these synapses drive this neuron to encode the digital spikes. If synapse dynamics affects signal integration and spike encoding at postsynaptic neurons [12], how does presynaptic transmitter release probability influence synaptic integration and then drive digital spike encoding at postsynaptic neurons? An essential solution to this issue is to quantify the correlations between release probability and neuronal encoding patterns (spike capacity and timing precision).

**Figure 5:**
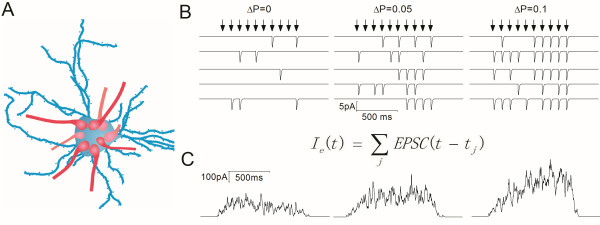
**A principle for the integration of synaptic inputs on postsynaptic neurons by computational simulation. A) **A neuron receives many synaptic inputs. **B) **These synapses in response to presynaptic manipulations possess the different changes in release probability (ΔP). The arrows present the firing of presynaptic spikes. **C) **shows the integrated current waveforms under the conditions of different release probabilities, based on the formula.

We investigated spike encodings at GABAergic neurons driven by glutamatergic synapses in distinct release probabilities. Figure 5 shows a principle of numerically computational simulation. Each neuron receives many synaptic inputs (Figure 5A), and each of synapses possesses the different changes of release probabilities under the different conditions (Figure 5B). In the simulation, we have taken the following factors into account, such as the glutamate contents released from individual vesicles, the probability of glutamate release, the number of release sites, the sensitivity of postsynaptic glutamate receptors, the number of synapses on a neuron as well as the input intervals from different presynaptic inputs. The values of glutamate release probability were given at different levels by introducing the averaged increments of release probability (ΔP) during sequential spikes from our data in Figures 1 and 2. Other parameters for the synapses are given in Methods (also [12,30]). Through such computational summations, we obtained the synaptic integrated current waveforms (Figure 5C).

**Figure 6:**
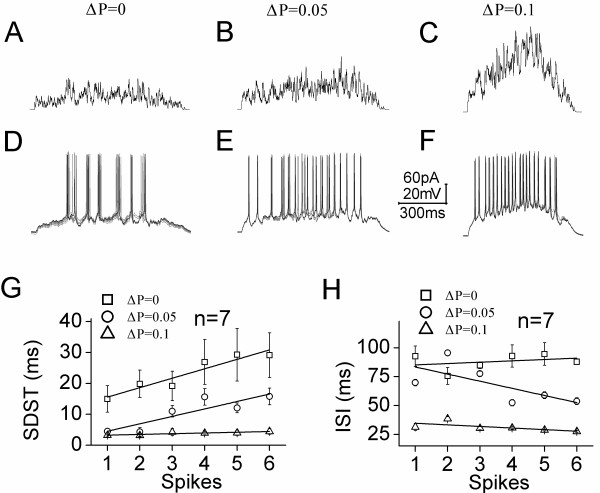
**The high increment of release probability during sequential presynaptic spikes enhances the capacity and precision of spike encodings at postsynaptic GABAergic cells. A-C) **show the integrated signals from unitary glutamatergic synapses under the conditions of release probability increments (ΔP) about 0, 0.05 and 0.1. **D-F) **show spike patterns induced by the integrated signals from unitary synapses under the conditions of release probability increments (ΔP) about 0, 0.05 and 0.1. **G) **shows the comparisons of standard deviation of spike timing (SDST) versus spikes under the conditions of release probability increments (ΔP) about 0 (square symbols), 0.05 (circles) and 0.1 (triangles, n = 7). **H) **shows the comparisons of inter-spike intervals (ISI) versus spikes under the conditions of release probability increments (ΔP) about 0 (square symbols), 0.05 (circles) and 0.1 (triangles). Calibration bars are 60 pA, 20 mV and 300 ms. Lines in **G ~ H **illustrate linear dynamical fitting.

We first examined the influences of probability increment on synaptic integrations and spike encodings, when the net probability increments (ΔP) were 0.05 and 0.1 caused by presynaptic spike frequencies at 10 Hz and 20 Hz, respectively. The control was set without probability increment, i.e., ΔP is equal to zero. The signals integrated from these synapses are showed in top panels of Figure 6 (6A for ΔP = 0, 6B for ΔP = 0.05 and 6 C for ΔP = 0.1).

These integrated signals were injected into postsynaptic neurons to examine their effects on spike encodings (n = 7). The spike patterns (Figure 6D) driven by the signals integrated from ΔP = 0 synapses (6A) appear not precise and reliable, compared with those spikes (Figure 6E ~ F) driven by the signals from ΔP = 0.05 (6B) and ΔP = 0.1 synapses (6C). The standard deviations of spike timing (SDST) are showed in Figure 6G. The values for SDST_1_ to SDST_6_ are 14.95 ± 4.3, 19.8 ± 4.45, 19.2 ± 4.79, 26.9 ± 7.3, 29.3 ± 8.53 and 29.1 ± 7.2 ms under a presynaptic ΔP = 0 (square symbols); the values are 4.49 ± 0.95, 4.32 ± 1.37, 11 ± 1.84, 15.6 ± 2.68, 12.1 ± 1.6 and 15.78 ± 2.68 ms under a ΔP = 0.05 (circles); and the values are 3.23 ± 0.28, 3.17 ± 0.5, 4.22 ± 1.1, 3.9 ± 0.54, 4.0 ± 0.85 and 4.43 ± 0.9 ms under a ΔP = 0.1 (triangles). SDST values for corresponding spikes under three conditions are statistically different (p < 0.01). Moreover, inter-spike intervals (ISI) are showed in Figure 6H. The values for ISI_1-2_ to ISI_6-7_ are 92.7 ± 8.7, 75.7 ± 7.3, 84.84 ± 2.92, 92.85 ± 9.6, 94.6 ± 9.8 and 88 ± 2.3 ms under a ΔP = 0 (square symbols); the values are 69.8 ± 2.76, 95.6 ± 2.4, 77.6 ± 2.16, 52.44 ± 2.74, 58.9 ± 3 and 53.7 ± 3 ms under a ΔP = 0.05 (circles); and the values are 31.44 ± 3.1, 38.5 ± 1.95, 30.4 ± 1.42, 30.64 ± 1.3, 28.84 ± 0.72 and 27.6 ± 0.96 ms under a ΔP = 0.1 (triangles). ISIs for corresponding spikes under three conditions are statistically different (p < 0.01). Therefore, the spike patterns driven by the synapses with release probability increment have high precision and capacity.

**Figure 7:**
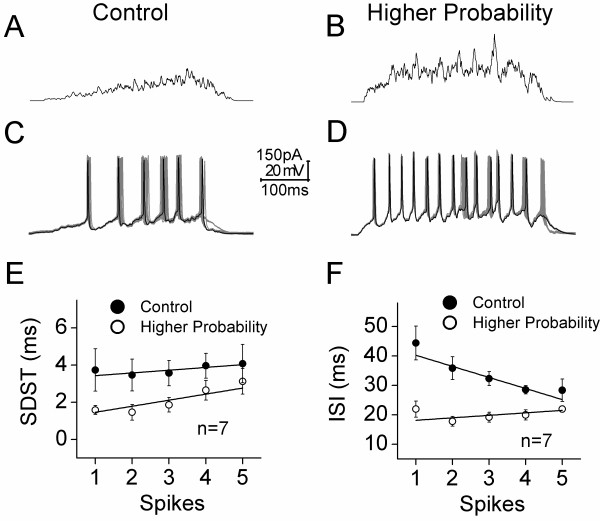
**Higher release probability during sequential presynaptic spikes enhances the capacity and precision of spike encodings at postsynaptic GABAergic neurons. A-B) **show the integrated signals from unitary glutamatergic synapses under the conditions of control (A) and adenophostin-A infusion (B, higher probability). **C-D) **illustrate spike patterns induced by the integrated signals from unitary glutamatergic synapses under the conditions of control (C) and AD infusion (D, higher probability) **G) **shows the comparisons of standard deviation of spike timing (SDST) vs. spikes under the conditions of control (filled symbols) and AD infusion (higher probability, opened symbols; n = 7). **H) **illustrates the comparisons of inter-spike intervals (ISI) vs. spikes under the conditions of control (filled symbols) and AD infusion (higher probability, opened symbols; n = 7). Calibration bars are 150 pA, 20 mV and 100 ms. Lines in **E ~ F **illustrate linear dynamical fitting.

We also examined the effects of probability increment on synaptic integration and spike encoding, when release probability was raised by elevating presynaptic basal Ca^2+^ (data in Figures 2D ~ F and 4C). The signals integrated from these synapses are showed in top panels of Figure 7 (7A for control, and 7B for higher release probability).

These integrated signals were injected into postsynaptic neurons to examine their effects on spike encodings (n = 7). The spike patterns (Figure 6D) driven by the signals integrated from ΔP = 0 synapses (6A) appear not precise and reliable, compared with those spikes (Figure 6E ~ F) driven by the signals from ΔP = 0.05 (6B) and ΔP = 0.1 synapses (6C). The standard deviations of spike timing (SDST) are showed in Figure 6G. The values for SDST_1_ to SDST_6_ are 14.95 ± 4.3, 19.8 ± 4.45, 19.2 ± 4.79, 26.9 ± 7.3, 29.3 ± 8.53 and 29.1 ± 7.2 ms under a presynaptic ΔP = 0 (square symbols); the values are 4.49 ± 0.95, 4.32 ± 1.37, 11 ± 1.84, 15.6 ± 2.68, 12.1 ± 1.6 and 15.78 ± 2.68 ms under a ΔP = 0.05 (circles); and the values are 3.23 ± 0.28, 3.17 ± 0.5, 4.22 ± 1.1, 3.9 ± 0.54, 4.0 ± 0.85 and 4.43 ± 0.9 ms under a ΔP = 0.1 (triangles). SDST values for corresponding spikes under three conditions are statistically different (p < 0.01). Moreover, inter-spike intervals (ISI) are showed in Figure 6H. The values for ISI_1-2_ to ISI_6-7_ are 92.7 ± 8.7, 75.7 ± 7.3, 84.84 ± 2.92, 92.85 ± 9.6, 94.6 ± 9.8 and 88 ± 2.3 ms under a ΔP = 0 (square symbols); the values are 69.8 ± 2.76, 95.6 ± 2.4, 77.6 ± 2.16, 52.44 ± 2.74, 58.9 ± 3 and 53.7 ± 3 ms under a ΔP = 0.05 (circles); and the values are 31.44 ± 3.1, 38.5 ± 1.95, 30.4 ± 1.42, 30.64 ± 1.3, 28.84 ± 0.72 and 27.6 ± 0.96 ms under a ΔP = 0.1 (triangles). ISIs for corresponding spikes under three conditions are statistically different (p < 0.01). Therefore, the spike patterns driven by the synapses with release probability increment have high precision and capacity.

We also examined the effects of probability increment on synaptic integration and spike encoding, when release probability was raised by elevating presynaptic basal Ca^2+^ (data in Figures 2D ~ F and 4C). The signals integrated from these synapses are showed in top panels of Figure 7 (7A for control, and 7B for higher release probability).

These integrated signals were injected into postsynaptic neurons to examine their effects on spike encoding (n = 7). Spike patterns (Figure 7C-D) driven by the signals integrated from the synapses appear less precise and reliable under control (7A) than higher probability (7B). SDST are showed in Figure 7E. The values for SDST_1_ to SDST_5_ are 3.73 ± 1.14, 3.47 ± 0.86, 3.57 ± 0.68, 3.97 ± 0.67 and 4.1 ± 1 ms under control (filled symbols); and the values are 1.59 ± 0.24, 1.47 ± 0.42, 1.86 ± 0.39, 2.65 ± 0.53 and 3.13 ± 0.7 ms under an elevation of presynaptic Ca^2+^ (opens). SDST values for corresponding spikes under these two conditions are statistically different (p < 0.01). In addition, their ISIs are showed in Figure 7F. Values for ISI_1-2_ to ISI_5-6_ are 44.35 ± 5.74, 35.8 ± 3.89, 32.28 ± 2.35, 28.5 ± 1.4 and 28.33 ± 3.83 ms under control (filled symbols); and their values are 21.95 ± 2.75, 17.76 ± 1.66, 19.2 ± 1.67, 19.96 ± 1.74 and 21.95 ± 1.1 ms under higher probability (opens). ISI values for corresponding spikes under these two conditions are statistically different (p < 0.01). Spike patterns driven by the synapses in higher release probability on GABAergic neurons are precisely reliable and highly capacity in a proportional manner.

**Figure 8:**
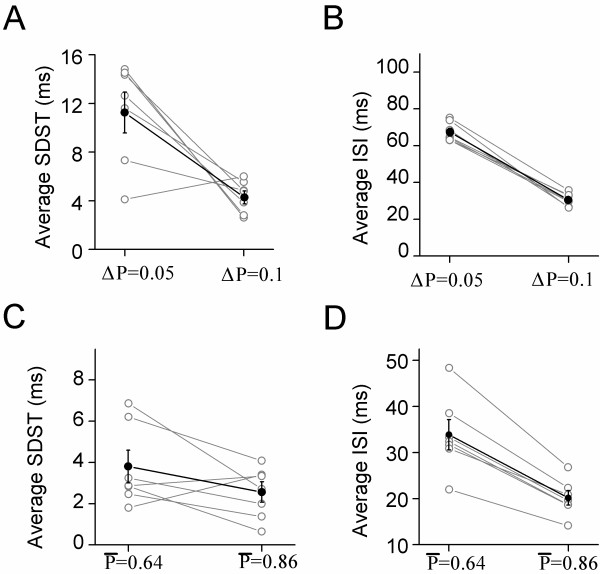
**Release probability increment and higher release probability enhance spike encodings at postsynaptic GABAergic neurons. A) **The higher increment of release probability during presynaptic sequential spikes reduces SDST. **B) **The higher increment of release probability during presynaptic sequential spikes reduces ISI. **C) **The higher release probability reduces SDST. **D) **The higher release probability reduces ISI.

To strengthen these indications above, we analyzed the quantitative changes of spike patterns and release probability (Figure 8). The probability increments from 0.05 to 0.1 make the averaged SDST to be changed from 11.34 ± 1.68 to 4.36 ± 0.53 ms (Figure 8A), and the averaged ISI from 67.45 ± 2.1 to 30.6 ± 1.42 ms (Figure 8B). The probability increases from 0.64 to 0.86 by elevating presynaptic Ca^2+^ make the averaged SDST to be changed from 3.76 ± 0.79 to 2.51 ± 0.5 ms (Figure 8C), and the averaged ISI from 35.85 ± 3.3 to 20.15 ± 1.59 ms (Figure 8D). Thus, presynaptic high release probability enhances the precision and capacity of postsynaptic neuronal encodings.

## Discussion

We investigated how the release probability of presynaptic glutamates quantitatively regulates synaptic dynamics and spike encoding at postsynaptic neurons. Presynaptic sequential spikes increase glutamate release probability and facilitate synaptic transmission in linearly correlated manner, which are Ca^2+^-dependent (Figures 1, 2, 3). The efficiency of probability-driven synaptic facilitation is upregulated by raising presynaptic Ca^2+^ levels (Figure 4). The elevation of glutamate release probability improves spike encoding at postsynaptic neurons (Figures 6, 7, 8). Our data indicate that the probability of releasing glutamate at excitatory synapses plays an important role in setting the encoding of digital spikes at postsynaptic neurons, i.e., high release probability strengthens the efficiencies of synaptic facilitation and of neuronal spike encoding in the brain networks. That is, the upregulation of glutamate release probability strengthens the conversion of synaptic analogue signals into neuronal digital spikes. The efficient and precise input signals to the neurons award them encoding digital spikes reliably, similar to memory retrieval and playback by a process that specific inputs induce precise outputs.

The probability of neurotransmitter release was regulated by presynaptic Ca^2+ ^[17-19,22,24,34-38] and other molecules [39-48]. Synaptic facilitation was mediated by Ca^2+^-dependent increments of transmitter release probability [49-55]. In addition to supporting these views about the association among presynaptic Ca^2+^, transmitter release probability and synaptic facilitation, we present the quantitative description for the correlations between release probability and synaptic facilitation when presynaptic neurons produce sequential spikes, especially these correlations are upregulated by elevating presynaptic Ca^2+^. That is, Ca^2+^-dependent probability increment is linearly correlated with synaptic facilitation. An increased release probability is more efficiently boosting synaptic facilitation (Figure 4).

In terms of the physiological significance of release probability increment, our results indicate that an increase of presynaptic release probability boosts the efficiency of probability-driven synaptic facilitation (Figure 4B ~ C), and in turn improves the capacity and timing precision of postsynaptic spikes (Figures 6,7,8). This enhanced spike encoding at postsynaptic neurons increases their release probability (Figure 1) and strengthens their downstream cells. By this functional compatibility between presynaptic and postsynaptic partners, the improvement of spike encodings at an individual neurons may circulate among network neurons and lead to the reliable output of brain codes for well-organized behaviors. It is noteworthy that each neuron sprouts many axon branches to innervate distinct postsynaptic neurons. It remains to be studied whether the pairs of presynaptic axon branches and postsynaptic neurons are functionally compatible. Based on the roles of release probability (Figures 6,7,8), uniform synaptic pattern [12] and quantal release [30] in spike encodings, we suggest that presynaptic quantal transmitters, release probability increment and postsynaptic receptor sensitization upregulate neuronal encoding coordinately. The reliable synaptic transmission drives precise neuronal encoding. It is noteworthy we provide experimental evidences for this point, although one could make such prediction. The weight of these synaptic factors on improving the postsynaptic spike encoding is under the study.

In mammalian’s brain, chemical synapses are dominant identities for signal communication among network neurons. The chain of neuron-synapse-neurons makes information flow in a sequence of action potentials, synaptic responsiveness and action potentials, i.e., digital-analogue-digital signals, different from digital signal flow through electrical synapses. The quantitative profiles underlying the conversions of digital-to-analog and analog-to-digital signals should be addressed to understand the programming of brain codes for well-organized cognition and behavior. Previous studies indicated the influence of presynaptic spikes on synaptic transmission patterns [18,19,22,24,36,37,49-58], which helps to understand the conversion of digital-to-analog signals. In addition to the regulation of postsynaptic receptors [12] and quantal transmitter release [30] to spike patterns, we present that high transmitter release probability enhances the efficiency of synaptic facilitation and neuronal encoding in the brain networks (Figures 4,5,6,7,8). These studies initiate a research field to address the quantitative conversions of digital-to-analogue and analogue-to-digital signals for a comprehensive picture about how the brain codes are programmed, in addition to the plasticity of synapses and neurons [59-67].

In synapse physiology, silent synapses are present in the central nervous system and involved in synaptic plasticity through their conversion [68,69]. The concept for silent synapse is established from the studies by giving a single stimulus, which may be due to the lack or low response of AMPA receptors [70-73]. When sequential spikes or multiple stimuli were induced in presynaptic neurons, the unitary glutamatergic synapses in no response to spike one or extremely low response probability were likely activated [72,74]; Figures 1 and 2). In this regard, the inactive synapses [72] and/or silent synapses [68,69] may result from a low probability of glutamate release at these synapses. The sequential spikes increase glutamate release probability at inactive synapses (Figures 1,2,3) and convert them into active ones, such that the number of active synapses may increased.

Glutamates are released in a quantal manner [14,21,22,30]. Their release probability and receptor responsiveness are changeable (Figures 1,2,3 and [12,57,58]. When the synapses transmit sequential spikes, their release probability and receptor responsiveness, compared with quantal release, are more likely responsible for the fluctuation and plasticity of synaptic activities. This point addresses an inconclusive view whether the fluctuations of synaptic strength under basal condition and plasticity result from the changes in the probability of transmitter release, the contents of released transmitter and/or the responsiveness of receptors [12,14,15,19,22,33,75-81].

## Method and Materials

### Brain slices

All experiments in our study were fully approved by Institutional Animal Care Unit Committee (IACUC) in Administration Office of Laboratory Animals Beijing China (B10831). Cortical slices (400 μm) were prepared from FVB-Tg(GadGFP)45704Swn/J mice whose GABAergic neurons in calretinin- and somatostatin-positive cells express green fluorescent protein (GFP). Mice in postnatal day 15–25 were anesthetized by injecting chloral hydrate (300 mg/kg) and decapitated by guillotine. The slices were cut by Vibratome in oxygenized (95% O_2_/5% CO_2_) artificial cerebrospinal fluid (mM: 124 NaCl, 3 KCl, 1.2 NaH_2_PO_4_, 26 NaHCO_3_, 0.5 CaCl_2_, 5 MgSO_4_, 10 dextrose and 5 HEPES; pH 7.35) at 4°C, and then were held in normal oxygenated ACSF (mM: 124 NaCl, 3 KCl, 1.2 NaH_2_PO_4_, 26 NaHCO_3_, 2.4 CaCl_2_, 1.3 MgSO_4_, 10 dextrose and 5 HEPES; pH 7.35) 24°C for 1–2 hours before the experiments. A slice was placed to submersion chamber (Warner RC-26 G) that was perfused with normal ACSF at 31°C for whole-cell recordings [82-84].

### The selection of pair-recorded neurons

Neurons in layers II ~ IV of sensorimotor cortex were selected for the pair-recordings. In synapse-coupled neurons, principal neurons had a pyramidal-like cell body and an apical dendrite; whereas GABAergic neurons appeared a round soma with multiple processes and GFP image (excitation 488 and emission 525) under a DIC and fluorescent microscope (Nikon FN-600). These pyramidal and GABAergic neurons demonstrated different responses to the hyperpolarization or depolarization pulses [72,84]. The rationales for using GABAergic neurons as postsynaptic neurons are the followings. Cortical pyramidal neurons have the extended dendrites, such as apical and basal dendrites; and GABAergic neurons possess the extended axonal arbors but not dendrites. Numerous synapses on pyramidal neurons are located on distal dendrites; and the synapses on interneurons are located closely to soma [12,85]. In order to prevent the effect of membrane cable properties on synaptic currents [86], we used GABAergic neurons as postsynaptic cells. In addition, GABAergic neurons are classified into different subtypes based on their protein markers, such as parvalbumin-, calbindin- and calretinin-positive neurons [87]. These Ca^2+^-binding proteins may cause their functional difference. To simplify the function of postsynaptic neurons in our study, we applied calretinin- and somatostatin- positive neurons (see above) as postsynaptic identities.

### Dual whole-cell recording

Single or multiple spikes in presynaptic pyramidal neurons were evoked by injecting depolarization pulses at 0.1 Hz. Pulse durations were 10 ms with an intensity to initiate single spikes in presynaptic neurons, which evokes mono-peak unitary excitatory postsynaptic current (uEPSC) at postsynaptic GABAergic neurons. Inter-pulse intervals to evoke the spikes were 50 ~ 100 ms. A MultiClamp-700B amplifier (Axon Instrument, Inc. Foster CA, USA) in current-clamp produced depolarization pulses to initiate presynaptic spikes. uEPSCs were recorded at GABAergic neurons in voltage-clamp model (holding potential, -70 mV). The electrical signals were inputted into pClamp-9 (Axon Instrument, Inc) for data acquisition and analysis. The transient capacitance was compensated, and output bandwidth was 3 kHz. Instantaneous and state-steady currents evoked by 5 mV pulses were monitored in all experiments, which were applied to calculate the series and input resistances. The probabilities of glutamate release during sequential spikes were calculated based on the counts of synaptic response versus failure. 10 μM 6-Cyano-7-nitroquinoxaline-2,3-(1 H,4 H)-dione (CNQX; Sigma, USA) was added after the end of experiments to examine GluR-mediated uEPSCs.

### The recordings of action potentials

Sequential spikes in GABAergic neurons were evoked by the signals integrated from the synapses in quantal release with the different probabilities of glutamate release. The integrated signals were converted into “abf” format for the interface with Clampex, and injected into these neurons by an amplifier (MultiClamp-700B) to evoke sequential spikes that were inputted into pClamp-9 for data acquisition and analysis. Input resistance is balanced, and output bandwidth is 4 kHz.

### Standard pipette solution for whole-cell recordings

It contained (mM) 150 K-gluconate, 5 NaCl, 0.4 EGTA, 4 Mg-ATP, 0.5 Tris-GTP and 4 Na-phosphocreatine, 10 HEPES (pH 7.4 adjusted by 2 M KOH). Fresh pipette solution was filtered with a 0.1 μm centrifuge filter before use. The osmolarity of pipette solution was 295–305 mOsmol. The resistance of pipettes was 8 ~ 10 MΩ to have a good access and prevent run-down in synaptic responses.

### The analyses of uEPSCs

Electrical signals were acquired by pClamp-9 via Digidata-1320A. uEPSCs in response to stimuli 1 ~ 8 were measured by Clampfit if the resting membrane potentials reached to −65 mV for GABAergic neurons. Data were analyzed if there were no significant changes in resting membrane potentials, action potentials and series/input resistances in all of the experiments. Indices in transmitter release patterns include the histograms of uEPSCs and the release probability versus spikes. The effects of release probability on synaptic patterns are presented by probability vs. uEPSC amplitudes. Data of uEPSCs induced among multiple pulses were statistically compared by one-way ANOVA. It is noteworthy that the responses of unitary synapses were analyzed before seeing the run-down in quantal sizes and their big variation (usually 20 ~ 25 min).

It is noteworthy that we use “normalized probability” to present changes of release probability based on the following thoughts. 1) The probability of initial responses is largely variable among the synapses. The variation of release probability is especially obvious under the conditions of synaptic plasticity. In order to statistically compare the net change in release probability among the synapses under the conditions of sequential spikes, adenophostin-A and BAPTA, we applied the “normalized probability”. 2) The normalized values of synaptic events were extensively used the study of synaptic plasticity, e.g., LTP and LTD. In these regards, we defined the release probability of initial response as P1 (numerical one), the release probability under the other conditions was calculated as the ratio of these probabilities to P1 (Pn/P1).

### Computational integrations of synaptic inputs

Signals from numerous inputs were integrated from excitatory synapses [12]. In the computational integrations for hundreds of presynaptic excitatory inputs that were activated randomly, presynaptic cells (j = 1, 2, …N) fired spikes at a specific rate, which evoked synaptic currents (i, i.e., uEPSCs) in a postsynaptic neuron at time t_1_, t_2_, ……t_n_. The integrated input currents (I) can be described.

$$I_{e}(t)=\sum_{j}EPSC(t-t_{j})$$

The integrated inputs were presumably correlated with uEPSCs, in which $EPSC(t)=A_{e}\frac{t}{\tau_{e}}e^{-t/\tau_{e}}$[88,89]. This was a simplified way to present the characteristics of low-pass filter in synaptic transmission in that currents were required to rise rapidly. In reality, the rising and decay phases of synaptic currents were slowly developed, and the synapses were driven by multiple presynaptic spikes. Therefore, we should apply the following kernel to present two sequential synaptic responses,

$$EPSC(t)=mte^{-t/\tau_{e}}\Theta (t)+n(t-T)e^{-(t-T)/\tau_{e}}\Theta (t-T)$$

in which *m* and *n* are the amplitudes of uEPSC one and two; and τ represents time constants. T is the time interval of inter-pulses at a synapse, and Θ(t) is Heaviside step function with Θ(t)=1 for t > 0, and Θ(t)=0 under other conditions.

The quantitative values used in the integration of currents from a population of glutamatergic unitary synapses on GABAergic cells are listed below. 1) The firing rate (F) of presynaptic pyramidal neurons is 17 Hz on average. 2) As asynchronously firing spikes in presynaptic neurons, inter-input intervals are 0.6 ~ 1.6 ms. 3) uEPSC1 ~ 5 amplitudes are 10.6 ± 2.1 pA (Figures 1 and 3). 4) The number of glutamatergic synapses on a postsynaptic cell is presumably 250 ~ 300. 5) The probability of releasing synaptic vesicles is a range of 0.2 ~ 0.5, based on the data in Figures 1, 2, 3, 4. The integration was done by self-program in Mat-lab.

## Competing interests

Author(s) declare that they have no competing interests.

## Authors’ contributions

JY and HQ contributed to the experiments and data-analyses. JHW took in charge of the experimental design and paper writing. All authors read and approved the final manuscript.
